# An irregular narrow complex tachycardia: atrial fibrillation or something else?

**DOI:** 10.1007/s12471-018-1216-z

**Published:** 2018-12-14

**Authors:** M. J. Mulder, C. P. Allaart, H. A. Hauer, M. J. B. Kemme

**Affiliations:** 1 Department of Cardiology, Amsterdam UMC, Amsterdam Cardiovascular Sciences, Vrije Universiteit Amsterdam, Amsterdam, The Netherlands; 2 Cardiology Centres of the Netherlands, Amsterdam, The Netherlands

A 44-year-old female patient was referred to our centre for pulmonary vein isolation. Her medical history was significant for hypothyroidism. She was taking levothyroxine and was clinically euthyroid with a free T4 of 19.9 pmol/l (12–22 pmol/l) and a thyroid-stimulating hormone level of 4.5 mU/l (0.3–4.5 mU/l). She experienced daily episodes of palpitations, not responding to antiarrhythmic drug therapy (sotalol or flecainide). Episodes were self-terminating within an hour of onset, without performing vagal manoeuvres. A 12-lead electrocardiogram was acquired during palpitations, which revealed an irregular narrow complex tachycardia that was previously diagnosed as atrial fibrillation (Fig. 1). Structural heart disease was excluded by transthoracic echocardiography. Based on this ECG an electrophysiology study was scheduled. During placement of the catheters, spontaneous initiation of an irregular tachycardia occurred (Fig. 2). What is the mechanism of this tachycardia?

**Fig. 1:**
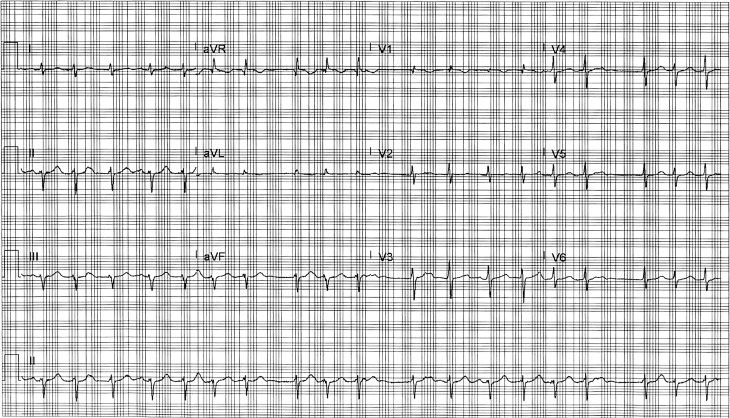
Electrocardiogram during palpitations

**Fig. 2:**
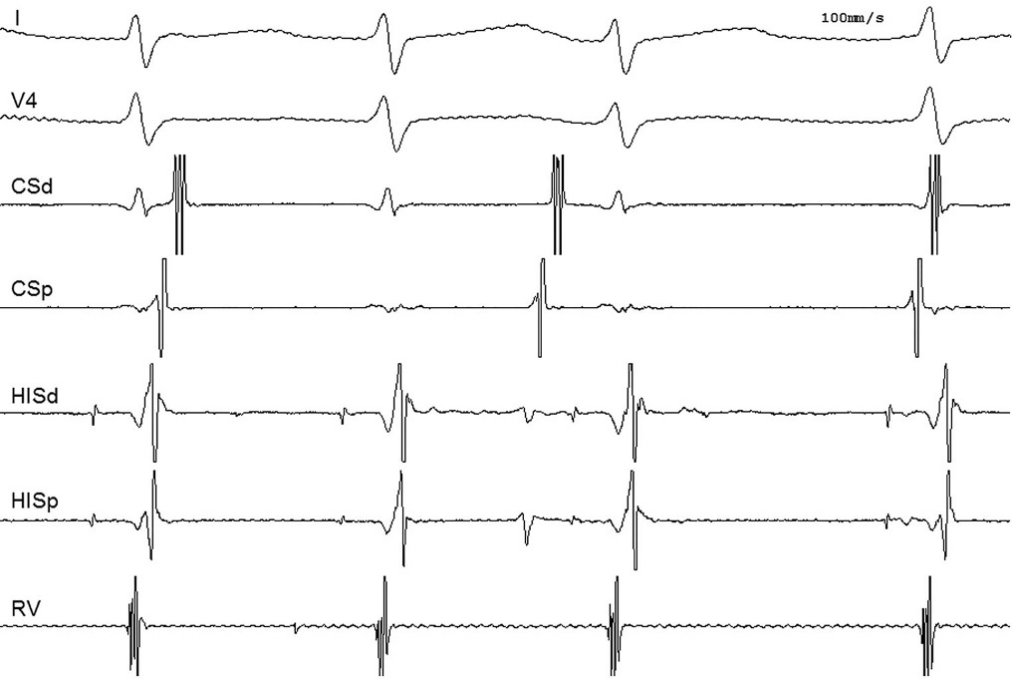
Tachycardia during electrophysiology study (*CSd* distal coronary sinus, *CSp* proximal coronary sinus, *HISd* distal His bundle, *HISp* proximal His bundle, *RV* right ventricle)

## Answer

You will find the answer elsewhere in this issue.

